# 

**Published:** 1868-12

**Authors:** 


### AMERICAN DENTAL ASSOCIATION—NOTICE.

Members of the American Dental Association who have not paid their annual dues for 1868, are requested to remit at once to the Treasurer, that he may be able to meet the requirements of the Publishing Committee in defraying the expenses of issuing the transactions. WM. H. GODDARD, Treasurer,

Corner of Fourth and Green Sts., Louisville, Ky.

October 22, 1868.

EDITOR DENTAL REGISTER: At the annual session of the Ohio State Dental Association, 1866, a committee was appointed to investigate the merits of the porcelain base, as improved by Dr. Dunn. The committee made a report, which was not published with the proceedings in the REGISTER. The following is the report, which you are requested to publish:

"Gentlemen of the Ohio State Dental Association:

"Your committee, to whom was referred the merits of the mineral or porcelain work manufactured and recently improved by Dr. W. E. Dunn, of Delaware, Ohio, have attended to that duty, and report:

"That they find that this style of work has undergone a complete change, not only in appearance, but in the manner of manipulating. It is now greatly simplified, and, as the teeth do not have to be carved, any one of ordinary ability can succeed with it and obtain good and artistic adaptation to the mouth. In fact, your committee are surprised at the great improvement in this work, and feel that the profession is greatly indebted to Dr. Dunn for his zeal and untiring efforts in this direction. Your committee is of the opinion that it is entitled to the favorable consideration of the profession, and that it should be adopted; and our investigations have determined us to adopt it in our own practice. Ordinary skill, such as will produce good results with other modes, will find little difficulty in manufacturing this style of work. Indeed, we think it will supersede other styles of work hitherto extensively employed.

"B. T. SPELLMAN,

"J. TAFT, } *Com.*"

"A. A. BLOUNT,

### CLOSE OF THE VOLUME.

With this number closes the twenty-second volume of the DENTAL REGISTER, the oldest Dental Journal in the world, except the *American Journal of Dental Science*, the publication of which was suspended for several years. The aim of the REGISTER has been to present to the profession that which is most valuable and interesting, both in regard to principles and practice, presenting such subjects as will best evolve the principles upon which correct practice is based. In regard to modes of practice, appliances, &c., the aim is to give that which is best; and especially to record any step of progress that is made. For the future, we have simply to say that the REGISTER will be, as it has been in the past, devoted to the best interests of t[illegible] [illegible]ental profession.

[illegible]

### LAWS TO REGULATE THE PRACTICE OF DENTISTRY.

It is a matter of much gratification to know that the profession in some of the neighboring States is moving in right good earnest, for obtaining laws to regulate the practice of Dentistry. Pennsylvania is moving in the right direction and with much energy. A State Society has there been formed, and its first work is to be devoted to this special object. This is the right method of taking hold of the matter. The interest of the profession should be as fully secured as possible. Indiana is also moving in the matter. The profession there has its State Society, through which it is operating for legal recognition. Michigan is also making a strong effort this winter, and we trust other States will soon take the matter in hand and do what may be done in this direction. Legislation to regulate the professions in this country is a new feature, the propriety of which has been called in question by many. It has been often objected that it is in opposition to the genius of our free institutions. A more mistaken idea, however, could hardly be entertained. The object of such laws, as it should be in all legislation, is simply the restraining and abatement of that which is injurious and wrong, and the encouragement and fostering of that which is right and productive of good. It is preposterous to assume that any one has the right, and should have the liberty to do that, for the faithful performance of which he is incompetent, and emphatically is this so, when the health and welfare of a fellow being is involved.

The law in Ohio has been in existence about nine months, and though only partially operative, and the time is yet too short to make any kind of a test of its efficiency, yet enough is already manifestly apparent, to indicate that it will be eminently efficient for good, though the law is, to a large extent, inoperative for four years to come. A number of cases could be referred to in which incompetent quackish pretenders have abandoned their business altogether, or gone to other States, where they could legitimately prey upon their unsuspecting victims.

We hope the professiou in our neighboring States will keep these gentry moving till they are in the "last ditch." T.

### New York College of Dentistry.

FACULTY.

ELEAZAR PARMLY, M.D., F.C.D.
Emeritus Professor of the Institutes of Dentistry.
WILLIAM H. DWINELLE, M.D.,
Professor of Dental Histology.
FRANK ABBOTT,
Professor of Operative Dentistry.
NORMAN W. KINGSLEY,
Professor of Dental Art and Mechanism.
SAMUEL R. PERCY, M.D.,
Professor of Dental Pathology and Therapeutics.
FANEUIL D. WEISSE, M.D.,
Professor of Descriptive and Comparative Anatomy, and Oral Surgery.
ALEX. W. STEIN, M.D.,
Professor of Experimental Physiology and Microscopic Anatomy.
P. H. VAN DER WEYDE, M.D.,
Professor of Chemistry and Metallurgy.

The plan of organization of this College provides for a course of instruction, didactic and demonstrative, by the above named Faculty and their Assistants in Practical Dentistry and collateral sciences; and also a Dental Infirmary, where clinical instruction will be given by the Board of Clinical Lecturers, which comprises a large nnmber of the most skillful gentlemen in the profession. This Board will give daily Clinics during the entire session. The advantages to the student of such a combination in acquiring a thorough and practical knowledge of the science and art, it is believed, cannot be excelled. The session will open on Thursday, Oct. 15th, 1868, and continue until the middle of March following.

Tickets for the course, $150, Matriculation, Demonstrators', and Diploma fees included. For further information, see College Announcement, or address the Dean of the Faculty.

NORMAN W. KINGSLEY, Dean,

No. 25 West 27th St., New York.

ELEAZAR PARMLY,

President Board of Trustees.

### PHILADELPHIA DENTAL COLLEGE.

108 North Tenth St., above Arch.

SIXTH ANNUAL SESSION, 1868-69.

FACULTY.

C. A. KINGSBURY, M.D., D.D.S.,
Professor of Operative Dentistry and Dental Histology.
J. H. McQUILLEN, M.D., D.D.S., Professor of Physiology.
ALBERT R. LEEDS, A. M., Professor of Chemistry.
WM. C. HEAD, D.D.S., Demonstrator of Operative Dentistry.
D. D. SMITH, D.D.S., Professor of Mechanical Dentistry and Metallurgy.
J. FOSTER FLAGG, D.D.S., Professor of Dental Pathology & Therapeutics.
HARRISON ALLEN, M.D., Professor of Anatomy and Surgery.
WM. P. HENRY, D.D.S., Demonstrator of Mechanical Dentistry.

The Dispensary and Laboratory of the College will be open and preliminary lectures delivered by one of the Professors, every day during the latter half of October, commencing on the 19th inst. The regular course of instruction will commence on the 1st of November, and continue until the close of the ensuing February. Every opportunity will be afforded in the Clinic and Laboratory for obtaining a *practical* knowledge of Operative and Mechanical Dentistry.

FEES.

| | |
|---|---|
| Matriculation (paid but once) - - - - - - - | $5 00 |
| Tickets for the *entire* Course, including the Demonstrators' | 100 00 |
| Diploma - - - - - - - - - - - | 30 00 |

For further particulars, address J. H. McQUILLEN, DEAN,
No. 1112 Arch Street, Philadelphia.

### BALTIMORE COLLEGE OF DENTAL SURGERY

TWENTY-NINTH ANNUAL SESSION, 1868–69.

### FACULTY.

THOMAS E. BOND, A.M., M.D., Prof. of Pathology and Therapeutics.
PHILIP H. AUSTEN, A.M., M.D., D.D.S., Prof. of Dental Science and Mechanism.
FERDINAND J. S. GORGAS, A.M., M.D., D.D.S., Prof, of Dental Surgery
A. SNOWDEN PIGGOT, A.M., M.D., Prof. of Chemistry and Metallurgy.
RUSSELL MURDOCH, A.M., M.D., Prof. of Anatomy.
HENRY REGINALD NOEL, A. M., M. D., Professor of Physiology.
HUGH McGINNIS GRANT, D.D.S., Demonstrator of Operative Dentistry.
THOMAS SOLLERS WATERS, D.D.S., Demonstrator of Mechanical Dentistry.
CLAUDE BAXLEY, M. D., Demonstrator of Anatomy.

The *Twenty-Ninth* Annual Session will commence on the fifteenth of October, 1868, and continue until March, 1869.

| | |
|---|---|
| Lecture and Demonstration Fees, - - - | $115 |
| Matriculation Fee (paid only once) - - - - - | 5 |
| Diploma Fee. - - - - - - - - | 30 |

Graduates of the Baltimore College of Dental Surgery can graduate at the Washington University of Medicine, Baltimore, on attending *one course* of Medical Lectures.

For information, address

F. J. S. GORGAS, M.D., Dean of the Faculty.

March '68-1y. *No.* 43 *Hanover St., Baltimore, Md.*

### The Dental Register

OF THE WEST.

*Issued Monthly, at $3 a Year in Advance.*

DEVOTED TO THE INTERESTS OF THE DENTAL PROFESSION.

EDITED BY J. TAFT AND G. WATT.

The volume commences in January and closes in December.

The Register being issued monthly is always fresh, therefore indispensable to the practitioner who desires to keep posted up with the new inventions and improvements which are daily developed. Neither expense nor effort will be spared to give value and variety to its contents. Articles will be illustrated with well executed engravings whenever they will add to the interest or assist in explanation.

Its extensive circulation and frequency of issue makes it one of the BEST DENTAL ADVERTISING MEDIUMS in the United States.

RATES OF ADVERTISING.

One page, 1 year, $50. Half page, 1 year, $30. Quarter page, or card, 1 year $20.

All advertising bills payable in advance, or at furthest after the issue of the first number containing the advertisement. All transient advertisements to be paid for in advance.

☞ CONTRIBUTIONS SOLICITED.

Address, J. TAFT, or "DENTAL REGISTER," Cincinnati Ohio.

Business Communications to

J. TAFT, Publisher,

No. 238 Fourth St., between Plum and Central Avenue

